# FAM83A is amplified and promotes tumorigenicity in non-small cell lung cancer via ERK and PI3K/Akt/mTOR pathways

**DOI:** 10.7150/ijms.33992

**Published:** 2020-03-12

**Authors:** Haiyang Hu, Fajiu Wang, Muyun Wang, Yuanyuan Liu, Han Wu, Xi Chen, Qiang Lin

**Affiliations:** 1 Department of Thoracic Surgery, Shanghai General Hospital, Shanghai Jiao Tong University School of Medicine, No. 100 Haining Road, Hongkou District, Shanghai 200080, China.; 2 Department of Thoracic Surgery, Hwa Mei Hospital, University of Chinese Academy of Sciences, No. 41 Xibei Road, Ningbo 315010, China.; 3 Department of Geriatric Respiratory and Critical Care, the First Affiliated Hospital of Anhui Medical University, No. 218 Jixi Road, Hefei 230022, China.; 4 Department of Otorhinolaryngology Surgery, Shanghai General Hospital, Shanghai Jiao Tong University School of Medicine, No. 100 Haining Road, Hongkou District, Shanghai 200080, China.; 5 Department of General Surgery, Shanghai General Hospital, Shanghai Jiao Tong University School of Medicine, No. 100 Haining Road, Hongkou District, Shanghai 200080, China.

**Keywords:** FAM83A, non-small cell lung cancer, tumorigenesis, metastasis

## Abstract

Family with sequence similarity 83A (FAM83A) is a newly-found over-expressed oncogene in several types of cancers and associates with poor prognosis. However, the role that FAM83A may play in the carcinogenesis of non-small cell lung cancer (NSCLC) still needs to be defined. The present study aimed to investigate the function of FAM83A in NSCLC progression and to investigate the possible mechanism. Analysis of Gene Expression Omnibus (GEO) database and rt-PCR showed up-regulated expression of FAM83A in NSCLC. GEO and the Cancer Genome Atlas (TCGA) data analysis revealed that high expression level of FAM83A in NSCLC was associated with poor prognosis. *In vitro* experiments showed that depleting FAM83A by siRNA/shRNA significantly inhibited cell proliferation and induced cell apoptosis. Cell motility was also retarded after silencing FAM83A, as demonstrated by Transwell assay. FAM83A depletion in A549 cells also inhibited subcutaneous tumor growth and lung metastasis *in vivo*. Western blotting showed that silencing FAM83A decreased the phosphorylation of ERK and PI3K/Akt/mTOR. On the other hand, overexpressing FAM83A *in vitro* enhanced cell proliferation and invasiveness, which was repressed by PI3K inhibitor and ERK inhibitor separately. Taken together, our study suggests that FAM83A promotes tumorigenesis of NSCLC at least partly via ERK and PI3K/Akt/mTOR pathways, making it a promising therapeutic target.

## Introduction

Lung cancer is leading cause of cancer-related morbidity and mortality across the world 1. Of all lung cancer cases, non-small cell lung cancer (NSCLC) is the most prevalent type and constitutes approximately 80% of lung cancer-related deaths 2. Despite of progress in the treatment of NSCLC over the past decades, currently available strategies including surgery, radiotherapy and chemotherapy have failed to satisfactorily improve the clinical outcome, owing to early metastasis of cancer cells 3. Nowadays targeted therapy has been routinely adopted in metastatic NSCLC patients with somatic sensitizing mutations such as EGFR, HER2 and KRAS. This has increased median overall survival among this patients group from 7.9 months on average in 2002 4 to 27.3 months in 2015 due to their apparent superiority in tumor response, progression-free survival (PFS), and life quality 5. Thus, identifying novel signaling molecules in carcinogenesis of NSCLC is crucial to finding effective therapeutic targets and improving survival.

Family with sequence similarity 83 (FAM83) oncogenes were discovered by two screens separately 6,7. Recently, multiple studies have identified overexpressed or dysregulated FAM83 members in cancer 8. Among the eight FAM83 family genes (FAM83A-H), the 434-amino acid FAM83A protein is the smallest one. It contains DUF1669, serine-rich domains, and prolinerich domains (PRDs). A conserved PxxP motif in the PRD domain interacts with Src homology 3 domain-containing proteins. A pseudo-PLD-like catalytic motif is contained in the DUF1669 domain, yet FAM83 proteins do not exhibit phospholipase activity 9,10. Recently it was found that the DUF1669 domain of FAM83 family proteins anchored casein kinase 1 isoforms which were implicated in the regulation of many cellular processes 11. FAM83A (originally named BJ-TSA-9) is highly expressed across tumor types 7,12,13. In pancreatic cancer, FAM83A is amplified and promotes cancer stem cell-like traits 12. In breast cancer cells, silencing FAM83A markedly decreased cell proliferation and invasion and elevated expression of apoptosis markers 7,14. Involved in a variety of important cancer cell signaling functions and overexpression in cancer, FAM83A is emerging as an intriguing oncogene worthy of additional study. However, the role that FAM83A may play in the carcinogenesis of NSCLC still needs to be defined, and the possible mechanism remains to be elucidated. In the present study, we investigated the significance of FAM83A in NSCLC, and preliminarily elucidated the possible mechanism through which FAM83A promoted cell proliferation and metastasis.

## Methods

### Cell culture

The human NSCLC cell lines (A549, H1299, H1581, and H460); human bronchial epithelial BEAS-2Bcells, and HEK293T cells were purchased from the cell bank of Shanghai Biology Institute, Chinese Academy of Science. All cell lines were authenticated by short tandem repeat fingerprinting and tested for mycoplasma contamination. According to American Type Culture Collection (ATCC, Manassas, VA, USA) protocols, cell lines and HEK293T cells were cultured in RPMI-1640 (Life Technologies, USA) or DMEM (Life Technologies, USA) at 37 °C in a humidified incubator under 5% CO_2_ conditions. Fetal bovine serum (10%) (FBS, Life Technologies) and 1% penicillin/streptomycin (Life Technologies) were supplemented into the culture medium. For inhibiting PI3K and ERK separately, wortmanin (Cayman, USA) and SCH772984 (MedChemExpress, USA) were used according to the manufacturers' protocols.

### Quantitative real-time PCR

Total RNA of cells was extracted with TRIzol reagent (Invitrogen, USA) according to the manufacturer's instructions. RNA was reverse transcribed using the PrimeScript RT Reagent Kit (Invitrogen, USA) and qPCR was performed using SYBR Premix Ex Taq (TaKaRa, Japan), following the manufacturer's instructions. The relative expression level of FAM83A gene was determined by the 2^-ΔCt^ method. GAPDH served as an internal control. Primers of FAM83A used for qRT-PCR assay are: forward: 5'-CCCATCTCAGTCACTGGCATT-3', reverse: 5'-CCGCCAACATCTCCTTGTTC-3'.

### Western blot analysis

Western blot analysis for determining protein expression was performed as described elsewhere 15. The antibodies used were as follows: anti-PCNA, anti-Bad, anti-Bax, anti-Bcl-xL, anti-p-ERK (Santa Cruz, USA), anti-MMP9, anti-p-PI3K p85, anti-p-Akt, anti-p-mTOR (Cell Signaling Technology, USA), anti-FAM83A (LifeSpan Biosciences, USA). Expression of Tubulin (Cell Signaling Technology, USA) was used as a protein loading control.

### Constructs and plasmids

The RNA duplexes for siRNA/shRNA-mediated FAM83A silencing were synthesized by Ribobio Company (Guangzhou, China). In addition, the plasmid of FAM83A was purchased from Ribobio Company. Transfections of the shRNA and overexpression vector in cells were performed using Lipofectamine 2000 Transfection Reagent (Invitrogen, USA) following the manufacturer's recommendation.

### CCK8 assay

Cells were seeded in 96-well plates at a density of 1 × 10^4^ cells/well and then incubated for 24, 48, and 72 hours. Following the manufacturer's instructions, cell viability was assessed using a Cell Counting Kit-8 (Dojindo Lab, Kumamoto, Japan). A microplate reader (Bio-Rad, USA) was used to measure the optical density values of each well at a wavelength of 450 nm.

### Clonogenic assay

Cells were plated at a density of 1 ×10^3^ cells per well in 6-well plates in DMEM/1640 medium plus 10% FBS and incubated at 37 °C for 10 days. Afterwards cells were stained with 0.2% methylene blue. Each well was photographed, and the number of colonies was counted.

### Fluorescence observation of cell apoptosis

To evaluate cell apoptosis after FAM83A depletion, Hoechst 33342 and propidium iodide (PI) staining were performed as described elsewhere 16.

### Wound healing assay

To evaluate motility of cells, wound healing assay was performed as previously described 17. Three different pictures were captured under an inverted microscope.

### *In vitro* migration and invasion assays

Transwell migration and invasion assays were performed as described previously 18. Samples were prepared in triplicate, and cells were counted on at least 3 different fields.

### Xenografts and tail vein injection assays

All procedures were conducted in accordance with National Institutes of Health guide for the care and use of Laboratory animals and conformed to our institutional ethical guidelines for animal experiments. Male BALB/c-nu mice (4-5 weeks old, 18-20 g) were purchased from Shanghai Laboratory Animal Company (SLAC, Shanghai). For subcutaneous implantation, cell suspensions (2 × 10^6^ cells) in a total volume of 100 μL were injected subcutaneously into the right flanks of nude mice. Tumor length and width were measured and recorded every 4 days starting 2 weeks after inoculation. Tumor volume was calculated as 1/2×length×width^2^. For lung metastatic mouse model, A549 cells (3 × 10^6^ cells in 0.2 mL PBS) were injected through the tail vein. The mice were sacrificed 6 weeks after the injection. The lungs were collected and paraffin embedded, and the hematoxylin and eosin (H&E) staining were performed using standard reagents and protocols 19.

### Statistics

All experiments were repeated at least three times with consistent results. Statistical analysis was performed using SPSS 19.0 (SPSS, Chicago, Illinois, USA). Differences between two groups were compared by Students' t test, and quantitative data were presented as mean ± SD. All tests of significance were two-sided and p < 0.05 was considered statistically significant.

## Results

### FAM83A is highly expressed in NSCLC and correlates with poorer prognosis

**Figure 1:**
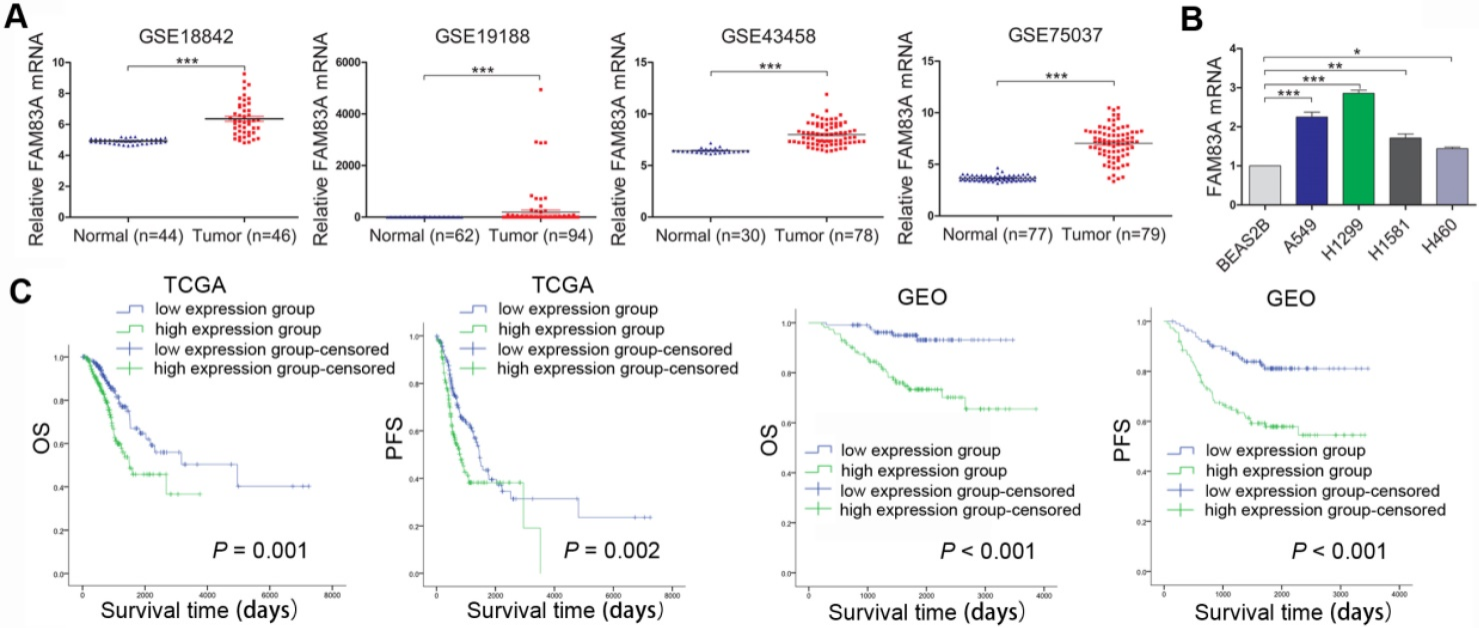
** FAM83A is overexpressed in NSCLC and correlates with poorer prognosis. (A)** Analysis of GEO database (GSE18842, GSE19188, GSE43458, GSE75037) shows higher expression level of FAM83A in NSCLC compared with normal tissues. **(B)** FAM83A mRNA expression in 4 NSCLC cell lines is higher than in normal bronchial epithelial cell line BEAS2B as tested by rt-PCR. **(C)** Prognosis analysis of TCGA and GEO databases shows that higher expression of FAM83A in NSCLC correlates with poorer overall survival and progression-free survival. *P < 0.05, **P < 0.01, ***P < 0.001.

Firstly, FAM83A expression level was analyzed in four multiple microarray datasets of NSCLC from Gene Expression Omnibus (GEO). As was demonstrated in Figure 1A, the GEO datasets (GSE18842, GSE19188, GSE43458, GSE75037) all showed that FAM83A expression level was significantly higher in NSCLC tissues than in normal tissues. The expression site of FAM83A was tested by immunocytochemistry, which presented cytosolic staining of FAM83A in A549 and H1299 cells, (Supplementary Figure 1, 100×). The FAM83A mRNA expression level was also investigated in NSCLC cell lines by rt-PCR. Compared with normal lung bronchial epithelial BEAS2B cell line, FAM83A is over-expressed in NSCLC cell lines A549, H1299, H1581 and H460 (Figure 1B). Moreover, prognosis analysis of TCGA and GEO databases indicated high expression of FAM83A in NSCLC correlated with poorer overall survival (OS) and progression-free survival (PFS) (Figure 1C).

### FAM83A promotes cell growth *in vitro*

**Figure 2:**
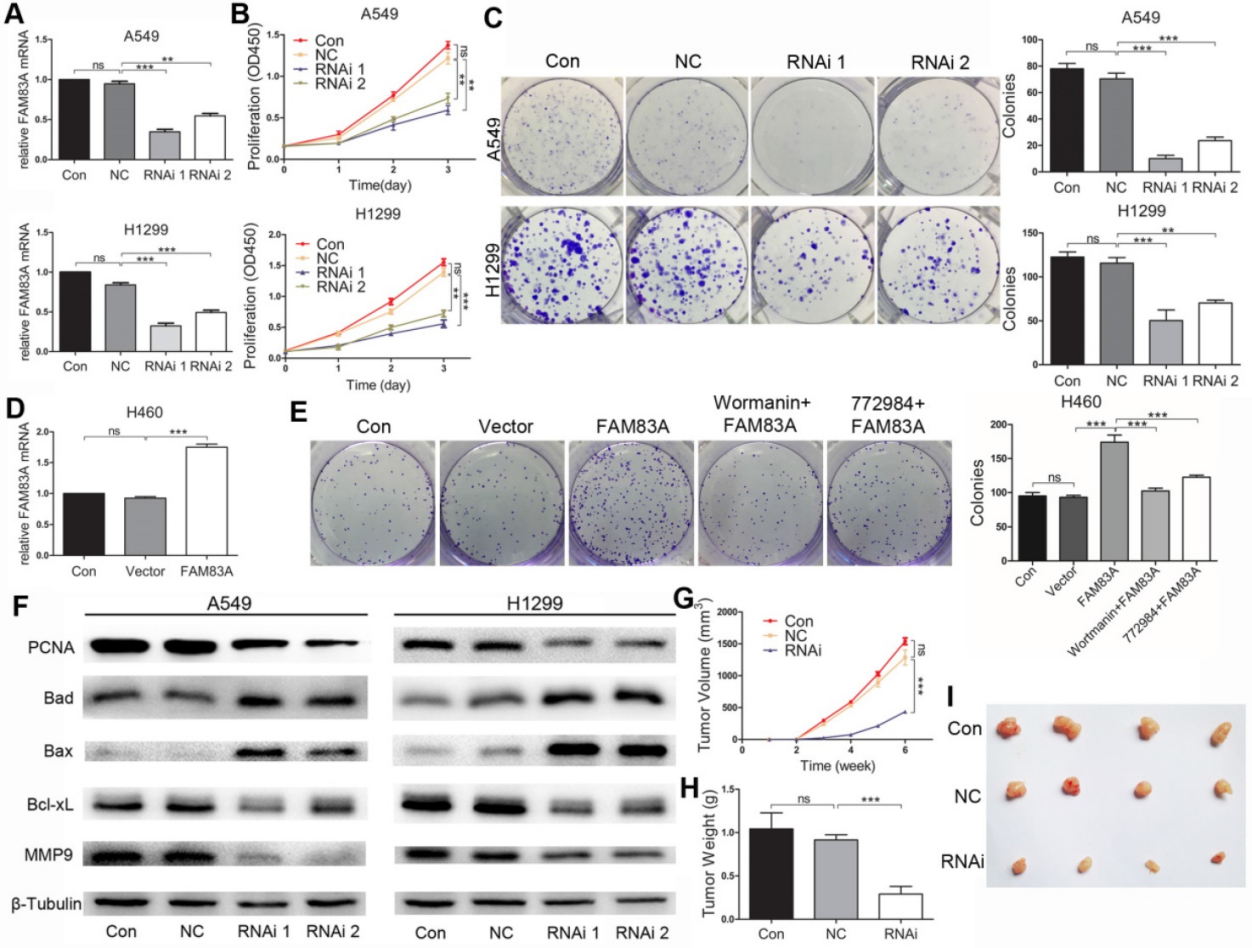
** FAM83A promotes NSCLC cell growth *in vitro* and *in vivo*. (A)** FAM83A is successfully depleted by siRNA in A549 and H1299 cells. **(B)** Knocking down FAM83A *in vitro* inhibits growth of A549 and H1299 cells as demonstrated by CCK8 assay. **(C)** Depleting FAM83A decreased number of clones formed in both A549 and H1299 cell lines. **(D)** FAM83A is successfully overexpressed in H460 cells. **(E)** Overexpressing FAM83A in H460 cells promotes clone formation, which is reversed by wortmanin and SCH772984. **(F)** Silencing of FAM83A decreases protein expression of PCNA, Bcl-xL, MMP-9 and increases expression of Bad and Bax. *In vivo* experiments shows FAM83A depletion in A549 inhibited subcutaneous tumor volume **(G)** and weight **(H)**, and representative figures are shown** (I)**. *P < 0.05, **P < 0.01, ***P < 0.001.

After depleting FAM83A by siRNAs in A549 and H1299 cells (Figure 2A), proliferation was significantly repressed compared with NC group at 3 time points (Figure 2B). Number of clones formed by the two cell lines in RNAi groups also decreased compared with NC group. Representative images are shown in Figure 2C. Western blot showed that silencing FAM83A inhibited expression of proliferation-related protein PCNA (Figure 2F). On the other hand, when FAM83A was overexpressed in H460 cells (Figure 2D), number of clones significantly increased (Figure 2E). Concordantly, overexpressing FAM83A in A549 cells promoted proliferation in CCK8 assay (Supplementary Figure 3A and B). WB results also demonstrated that silencing FAM83A decreased the protein expression level of proliferation-related PCNA (Figure 2F). Furthermore, FAM83A ablation in A549 cells significantly inhibited subcutaneous tumor growth *in vivo* (Figure 2G). Tumor weight was also repressed in RNAi group compared to NC group (Figure 2H). Images of tumors are shown in Figure 2I.

### FAM83A knockdown rendered cells to apoptosis

**Figure 3:**
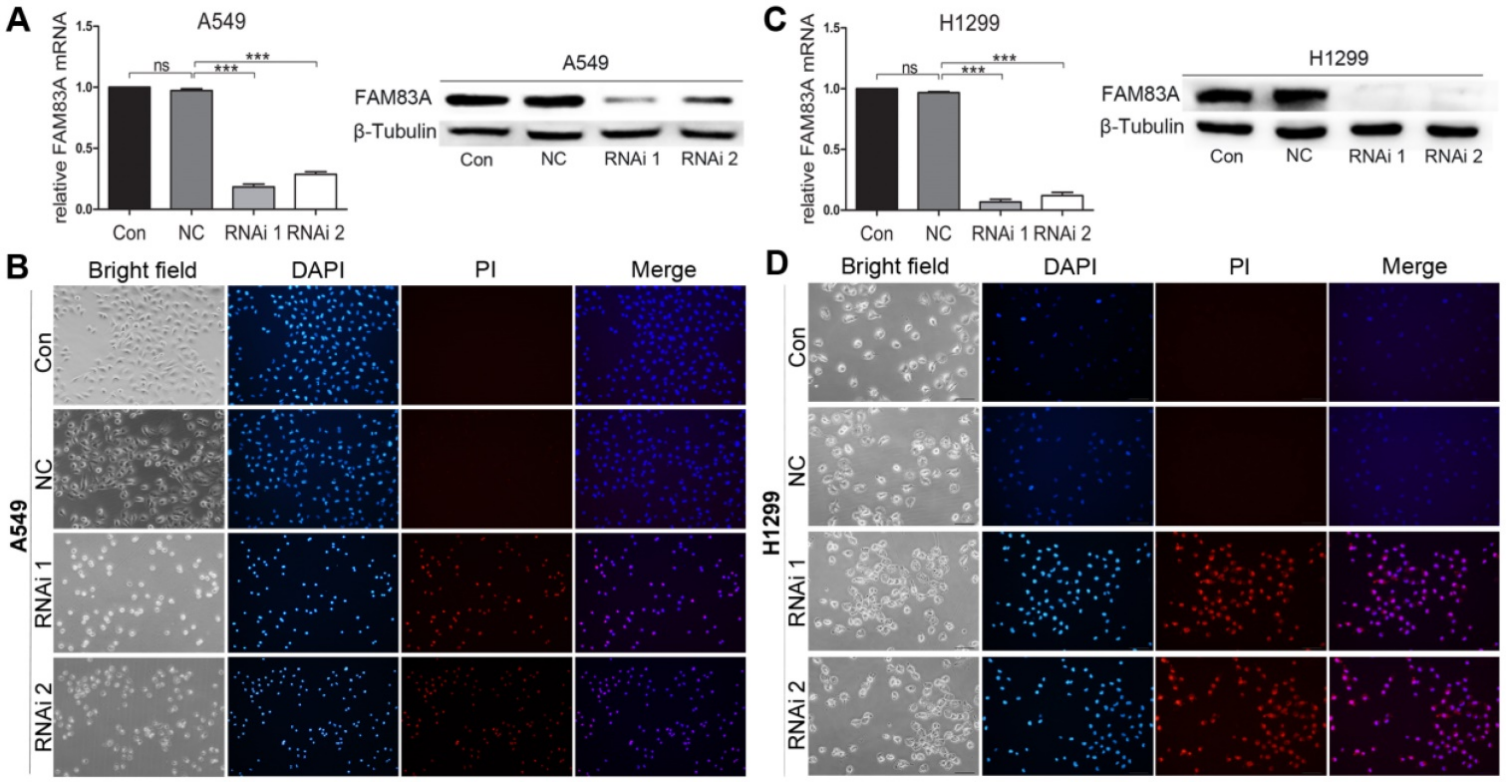
Depleting FAM83A promotes NSCLC cell apoptosis. **(A, C)** Rt-PCR and WB show that FAM83A is successfully knocked down by 2 shRNAs in A549 and H1299 cells. **(B, D)** Cell apoptosis is induced by specified concentration of H_2_O_2_ and stained by PI for detection of apoptosis. RNAi groups show significant cell apoptosis compared with Con and NC groups (Magnification: 200×).

Two shRNAs targeting human FAM83A and a non-specific scramble shRNA sequence (NC) were cloned into a lentiviral vector. Then the lentiviruses were produced to infect A549 and H1299 cells for FAM83A knockdown. Both shRNA viruses efficiently suppressed FAM83A expression in cells (Figure 3A and C). Cells in Control and NC groups didn't show typical apoptosis sign (Figure 3B and D, upper panels), but obvious apoptosis of A549 and H1299 cells in RNAi groups was observed as dyed red by PI (Figure 3B and D, lower panels). WB demonstrated that silencing FAM83A increased protein expression of pro-apoptotic Bad and Bax while decreased expression of anti-apoptotic Bcl-xL (Figure 2F). Pro- and anti- apoptotic protein expression were also measured *in vivo*. After FAM83A was silenced, expression of pro-apoptotic Bad and Bax were increased anti-apoptotic Bcl-xL was decreased (Supplementary Figure 2). The results indicated an anti-apoptotic role of FAM83A in NSCLC.

### FAM83A facilitated NSCLC metastasis

**Figure 4:**
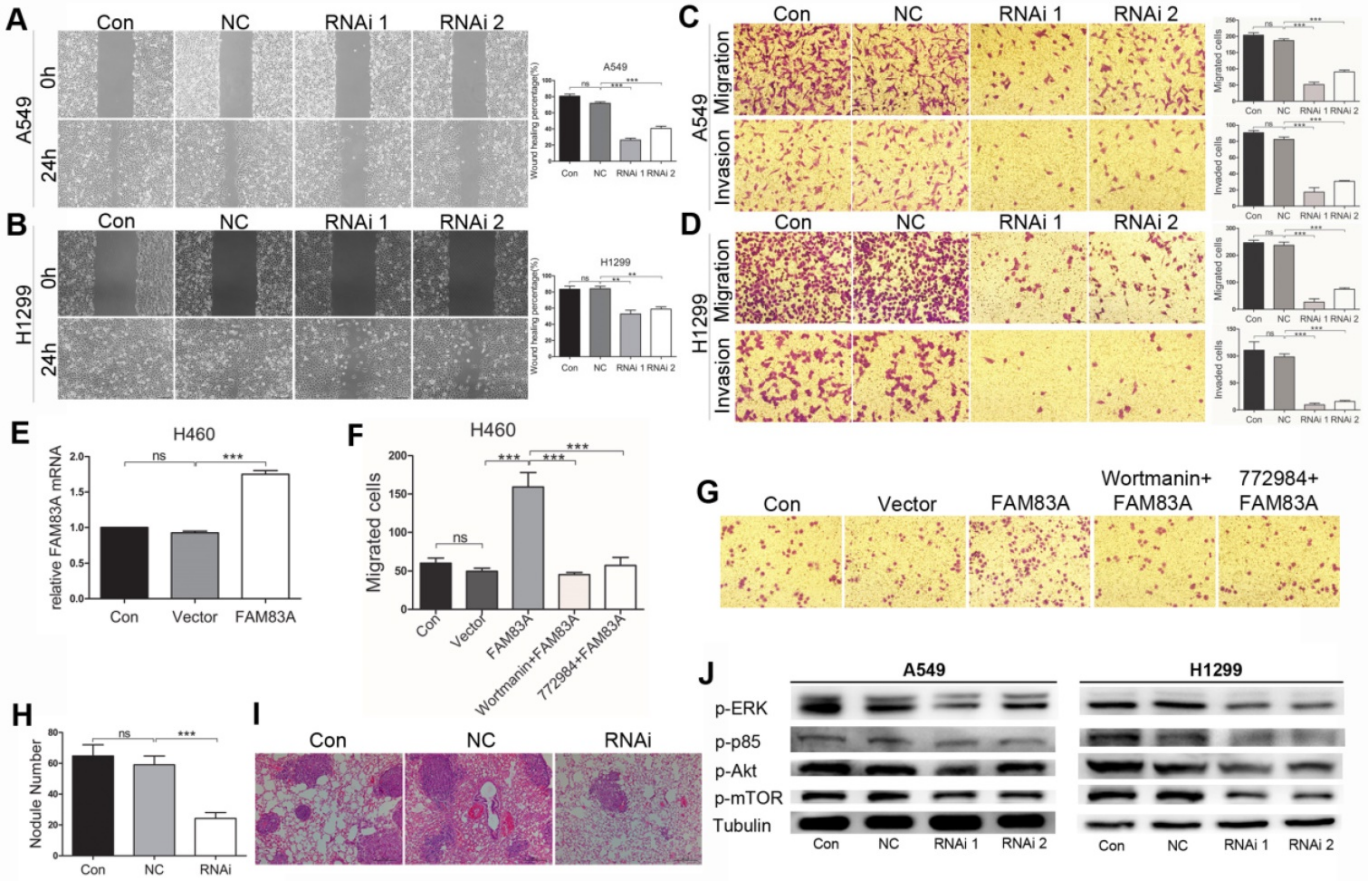
FAM83A promotes NSCLC metastasis and functions through ERK and PI3K/Akt/mTOR pathways. **(A, B)** Depleting FAM83A in A549 and H1299 cells restrains wound healing percentage in Scratch assay (Magnification: 200×). **(C, D)** Depleting FAM83A in A549 and H1299 cells decreases number of migrated and invaded cells in Transwell assays (Magnification: 200×). **(E)** FAM83A is overexpressed in H460 cells. **(F)** Overexpressing FAM83A increases number of migrated H460 cells in Transwell assay, which is reversed by wortmanin and SCH772984. **(G)** Representative images of H460 Transwell assay are shown (Magnification: 200×). Lung metastasis model of mice is established by tail vein injection of A549 cells. RNAi group shows less metastatic nodules on the lung surface** (H)**, which is also illustrated in lung sections (**I,** magnification: 200×). **(J)** WB shows that depleting FAM83A in A549 and H1299 cells mitigated protein expression of p-ERK, p-PI3K p85, p-Akt and p-mTOR. **P < 0.01, ***P < 0.001.

As an important cancerous feature, cell metastasis was evaluated *in vitro* after silencing FAM83A in A549 and H1299 cells. Firstly, wound scratch assay was conducted. After an even scratch by a 10 μL tip and incubated for 24h, the remaining cells could migrate to close the wound. As was shown in Figure 4A and B, silencing FAM83A diminished healing percentage compared with NC groups. Transwell assays were performed afterwards. The number of cells that migrated through the 8μm-pored membrane to the lower side of Transwell showed no significance in Control group and that in NC group. Yet after silencing FAM83A, number of migrated cells remarkably decreased in RNAi groups than in NC group (Figure 4 C and D upper panels). In Transwell invasion assay, number of invaded cells in RNAi groups also significantly reduced compared to NC groups (Figure 4C and D, lower panels). WB showed that FAM83A knockdown significantly decreased the expression of MMP-9 in both cell types (Figure 2F). On the other hand, overexpressing FAM83A in H460 cells promoted cell migration (Figure 4E and F). Representative images were shown in Figure 4G. Concordantly, overexpressing FAM83A promoted cell motility in A549 cells (Supplementary Figure 3C and D). Furthermore, to evaluate the role of FAM83A in metastasis *in vivo*, a mouse model of hematogenous tumor metastasis was established by injecting A549 cells through the tail vain. After mice were sacrificed, the number of metastatic nodules at the lung surface was counted and the right lungs were sectioned and underwent H.E staining. Compared to Control and NC groups, number of nodules at the lung surface in RNAi group significantly reduced (Figure 4H). Representative images of metastatic foci in lung sections are shown in Figure 4I.

### FAM83A promoted NSCLC via ERK and PI3K/Akt/mTOR signaling pathways

As ERK and PI3K/Akt/mTOR signalings are closely correlated with tumor growth and metastasis, we tested whether FAM83A exerted its function via these two pathways. As was shown by WB in Figure 4J, silencing FAM83A decreased expression of p-ERK, p-p85, p-Akt and p-mTOR in A549 and H1299 cells. Overexpressing FAM83A in H460 and A549 cells promoted cell growh, which was retarded by PI3K inhibitor (wortmanin) and ERK inhibitor (SCH772984) (Figure 2E and supplementary Figure 3B). FAM83A over-expression also increased number of migrated H460 and A549 cells in Transwell assay, which was partly repressed by PI3K inhibitor (wortmanin) and ERK inhibitor (SCH772984) (Figure 4F and G, supplementary Figure 3C and D). The results collectively demonstrated that FAM83A promoted NSCLC proliferation and metastasis at least partly via ERK and PI3K/Akt/mTOR signaling pathways.

## Discussion

The findings of the present study shine a spotlight on the potential oncogenic role of FAM83A in NSCLC progression. We demonstrate that FAM83A is significantly overexpressed in NSCLC and its high expression profile correlates with poor prognosis. Moreover, we find that FAM83A promotes lung cancer progression through ERK and PI3K/Akt/mTOR pathways, suggesting this protein has potential as a therapeutic target for NSCLC.

Cell signaling networks are complex. Intercommunicating pathways work in alliance to regulate cellular functions. If these tightly controlled pathways are disrupted, however, cells may become cancerous 20. In the present study, we found that FAM83A exhibited high expression level in NSCLC, which was identified by analysis of GEO database and by rtPCR in NSCLC cell lines. Data from TCGA and GEO also revealed an association between FAM83A overexpression and poor prognosis of patients with NSCLC. Concordantly, researches proved that FAM83A presented high expression in several other human cancers, such as in breast, testis, bladder and pancreatic cancer 12,21,22. Moreover, high expression level of FAM83A in pancreatic cancer was correlated with poorer survival 12. When exploring the mechanism of FAM83A up-regulation, its genomic locus is of interest. FAM83A gene is located on chromosome 8q24 23. This region is known to contain the oncogene Myc, which is frequently amplified in cancer 24. It has also been recently proved that long non-coding RNA (lncRNA) FAM83A-AS1 promotes lung adenocarcinoma by increasing FAM83A expression 25. In another research, the expression of FAM83A was showed to be influenced by EGFR levels, pathway signaling, and mutation status 26. Together with these findings, we believed that FAM83A may play an important role in promoting NSCLC progression. The hypothesis was subsequently validated *in vitro* and *in vivo*.

Firstly, we examined the function FAM83A may exert in NSCLC by silencing the expression of FAM83A in two NSCLC cell lines. FAM83A depletion in cultured A549 and H1299 cells reversed the malignant phenotype. After FAM83A was knocked down by siRNAs, compared with the Control and NC groups, cell proliferation in RNAi group was repressed and apoptosis was induced in cells treated with H_2_O_2_. Also, depleting FAM83A decreased the expression of proliferating cell nuclear antigen (PCNA) and Bcl-2, and increased expression of Bad and Bax. Migration and invasion were retarded after silencing FAM83A by shRNA in cells and MMP-9 expression level was inhibited. On the other hand, overexpressing FAM83A in H460 cells facilitated tumor proliferation and migration, as was demonstrated by clone formation and Transwell assays. Moreover, we identified the promoting effect of FAM83A on NSCLC in animal experiments. Compared with the Control groups, silencing FAM83A in A549 cells both inhibited subcutaneous tumor growth and hematogenous metastasis in mice. These data demonstrated an association of FAM83A with lung neoplasia.

Afterwards we investigated the possible mechanism through which FAM83A may promote NSCLC progression. PI3K/AKT/mTOR kinases are important regulators of multiple cellular processes, including metabolism, proliferation, protein synthesis, programmed cell death, tumor invasion and angiogenesis 3. Deregulated activation of AKT/mTOR occurs in 70% of cases of NSCLC. Thus, aberrantly-activated AKT/mTOR is a relevant therapeutic target in lung cancer. Unregulated ERK signaling also plays a vital role in tumor growth 27 and metastasis 28. In the present study, the expression level of p-p85, p-Akt, p-mTOR and p-ERK all decreased after FAM83A depletion. As well, over-expressing FAM83A in H460 cells promoted proliferation and motility, which was repressed by wortmanin and SCH772984. The results all demonstrated that FAM83A promoted NSCLC progression at least partly via PI3K/Akt/mTOR and ERK pathways. Accordingly, Lee et al. 7 also identified that silencing FAM83A in breast cancer cells markedly resulted in decreased p-ERK and p-AKT expression while suppressing malignant phenotype both *in vitro* and *in vivo*.

The phosphorylation of FAM83A is also an intriguing issue. In cancer, aberrant phosphorylation of signaling effectors, typically by receptor tyrosine kinases, drives unchecked proliferation and survival. Previous studies indicated phosphorylation of FAM83A is crucial for its signaling function 7,29. The Bose group found that FAM83A in HER2-positive breast cancer was hyper-tyrosine phosphorylated 29. However, the kinases that phosphorylate FAM83A remain unknown. The Bissell group showed that FAM83A tyrosine phosphorylation increases immediately upon EGFR activation, implying that EGFR may directly phosphorylate FAM83A 7. Additional work will be needed to identify the role of FAM83A phosphorylation regulating signaling complex formation and in FAM83A-mediated transformation.

## Supplementary Material

Supplementary figures and tables.

## Abbreviations

FAM83A: Family with sequence similarity 83A
NSCLC: non-small cell lung cancer
GEO: Gene Expression Omnibus
TCGA: the Cancer Genome Atlas
PFS: progression-free survival
PRDs: prolinerich domains
OS: overall survival
