# Supplementary material for: FAM83A is amplified and promotes tumorigenicity in non-small cell lung cancer via ERK and PI3K/Akt/mTOR pathways
Source: Int J Med Sci. 2020 Mar 12;17(6):807–14. doi: 10.7150/ijms.33992 (PMC7085261; doi:10.7150/ijms.33992)

Figure Legends

Supplementary Figure 1: FAM83A shows cytosolic staining in A549 and H1299 cells by immunocytochemistry (100×).

Supplementary Figure 2: Silencing FAM83A promots apoptosis in vivo. Pro- and anti- apoptotic protein expression are measured by western blot. After FAM83A is silenced, expression of pro-apoptotic Bad and Bax increased but no positive result is showed in Control and NC groups. Meanwhile, expression of anti-apoptotic Bcl-xL is decreased after FAM83A was silenced.

Supplementary Figure 3: FAM83A promotes proliferation and cell invasion in A549 cells through ERK and PI3K/Akt/mTOR pathways. After FAM83A is overexpressed in A549 cells (A), proliferation of A548 cells in CCK8 assay is promoted (B) and cell invasion in Transwell assay is facilitated(C and D) (Magnification: 200×), which are partly reversed by wortmanin and SCH772984.

Supplementary Figure 1:

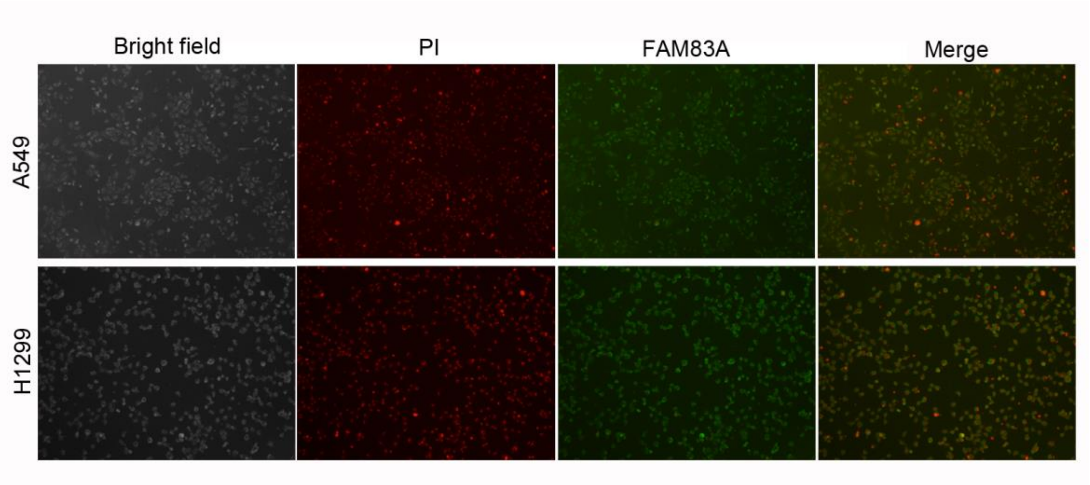

Supplementary Figure 2:

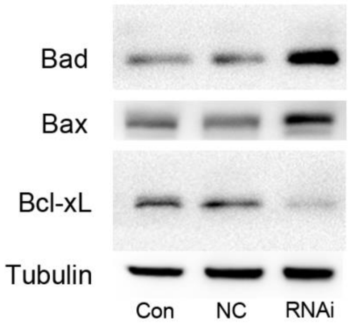

Supplementary Figure 3:

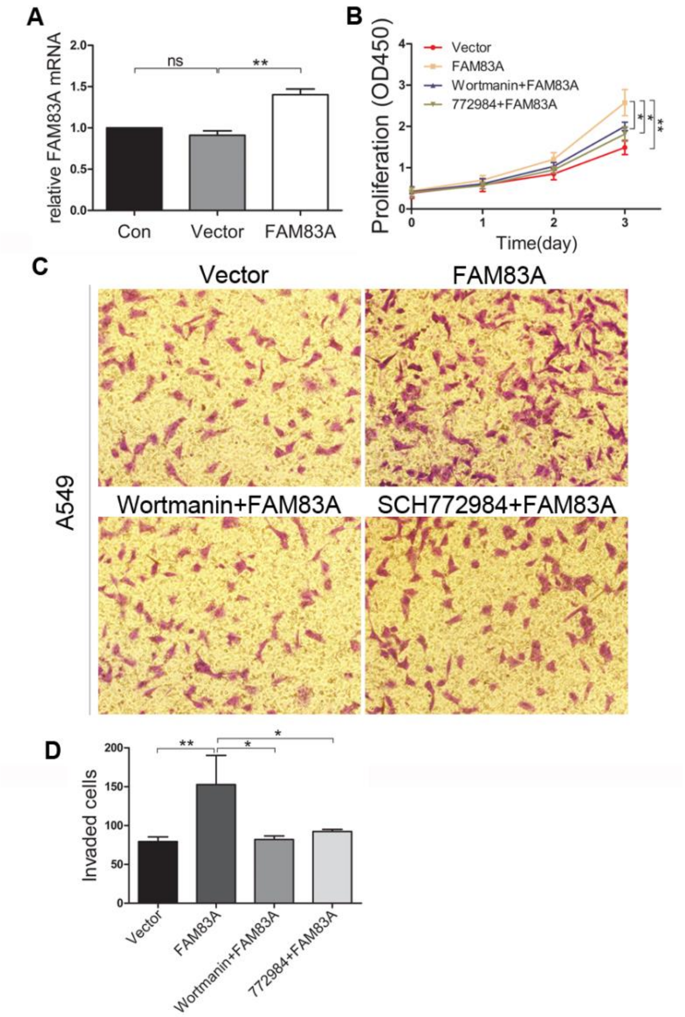

Supplement: Supplementary file 1 — Supplementary figures and tables. [file ijmsv17p0807s1.pdf]
